# Assessment of Hammer Energy Measurement for the Standard Penetration Test (SPT) Using Pile Driving Analyzer and Kallpa Analyzer Devices in Peru

**DOI:** 10.3390/s25051460

**Published:** 2025-02-27

**Authors:** Carmen Ortiz, Jorge Alva, José Oliden, Nelly Huarcaya, Grover Riveros, Roberto Raucana

**Affiliations:** 1 Research Center for Digital Transformation in Engineering, Universidad Nacional de Ingeniería, Lima 15333, Peru; jalvah@uni.edu.pe (J.A.); joliden@uni.edu.pe (J.O.); nhuarcaya@uni.edu.pe (N.H.); groveross@uni.pe (G.R.); rraucanas@uni.pe (R.R.); 2 Faculty of Engineering, Universidad Peruana de Ciencias Aplicadas, Lima 15023, Peru; 3 GERDIS Group, Civil Engineering Division, Pontificia Universidad Católica del Perú, Lima 15088, Peru

**Keywords:** energy measurement, standard penetration test (SPT), Kallpa, Pile Driving Analyzer (PDA), N_60_, relative density of soil

## Abstract

Energy measurement in dynamic penetration tests is key to correctly interpreting test results and ensuring comparable geotechnical data. Although commercial devices are widely used, their high cost limits adoption in developing regions such as Peru, affecting the accuracy of soil evaluation in many geotechnical studies. In this context, this research presents an energy measurement system called Kallpa, which uses low-cost electronic components to digitize sensor signals during Standard Penetration Tests (SPTs). Kallpa employs high-resolution analog-to-digital converters (ADCs) with an advanced sampling frequency, processing and storing data via a Raspberry Pi 4 microcomputer. The sensors, including accelerometers and strain gauges, were calibrated and compared with the Pile Driving Analyzer (PDA) to validate their accuracy in the Kallpa system. This study involved sixteen Standard Penetration Tests (SPTs) conducted in various regions of Peru using donut hammers and two tests involving automatic hammers. The results demonstrate that the Kallpa system is comparable to other energy measurement devices on the market, such as the Dynamic Penetration Test (DPT), which provides accurate SPT energy measurements. The Kallpa Processor (Version 1.0) software was developed to perform data acquisition and calibration, analyzing approximately 500 hammer blows and comparing peak values with those of the Pile Driving Analyzer. The data collected by Kallpa’s sensors strongly agreed with the PDA data, validating the reliability of the device. The Energy Transfer Ratio (ETR) for manual hammers ranged from 43.5% to 68.4%, with an average of 58.9%, whereas automatic hammers presented ETR values between 82% and 87%. The correction of the N60 blow count allowed for the estimation of the relative density of soils evaluated at different depths and locations across Peru.

## 1. Introduction

The Standard Penetration Test (SPT) stands out among other soil investigation methods as one of the most widely used techniques worldwide for obtaining geotechnical parameters. In Peru, more than 70% of geotechnical companies employ the SPT, highlighting the importance of this research in reducing measurement errors. Although other tests, such as the Cone Penetration Test (CPT), offer greater accuracy and a more detailed characterization of the soil, their application in Peru is limited due to high implementation costs. In contrast, the SPT is widely used due to its lower cost and the availability of numerous empirical correlations.

The popularity of the SPT is based on its practicality, lower costs, the extensive experience accumulated by other researchers, and the ease of interpreting its results. Consequently, the N value obtained from the test has become a fundamental index for soil characterization, influencing foundation design, seismic assessments, and other geotechnical analyses.

Since its inception and its first standardization by the American Society for Testing and Materials (ASTM) in 1958 [1], the procedures and types of samplers, along with penetration measurements, have been detailed. However, internationally, there is consensus on the importance of adopting the “Reference Test Procedure”. This standardization of procedures has generated interest in evaluating the test’s efficiency. Although it is widely used globally, the lack of uniformity has led to research on the measurement of hammer drop energy.

Over time, various studies have sought to identify the influence of different factors on penetration measurements. However, the variability in SPT N values makes establishing a representative value for the strata difficult.

The importance of measuring energy stems from its direct influence on penetration resistance values. Variability in SPT N values can result from differences in hammer drop mechanisms, rod alignment, and the efficiency of energy transfer. Studies by researchers such as Meyerhof [2], Kanai [3], Teng [4], Shibata [5], Seed and Idriss [6], Bowles [7], Dikmen [8], Fauzi [9] and Panjamani et al. [10] have estimated geotechnical parameters using the SPT N value. However, variations in these measured values complicate the accurate representation of soil strata, highlighting the necessity of standardizing energy transfer evaluations.

These limitations have driven research on measuring hammer drop energy, from the pioneering works of Palacios and Schmertmann [11,12] and Kovacs [13] to the contributions of Kovacs [14], Matsumoto [15], Odebrecht et al. [16], Aoki and Cintra [17], Jong-Sub Lee and Yong-Hoon Byun [18], Zhang et al. [19], and Hong et al. [20]. These studies identified the influence of various factors on penetration measurement, particularly the reduction in impact energy in the hammer lifting and release systems. This divergence leads to the transfer of different energies to the rods, resulting in divergent outcomes.

Globally, methods have been developed to measure the energy transmitted to rods, and these methods were standardized in 1986 [21]. This has led to a reduction in the variability of penetration resistance results. To address this situation, companies and researchers have designed energy measurement instruments, with the Pile Dynamics PDA analyzer being one of the most recognized instruments worldwide.

Currently, there are few devices specifically designed for energy measurement in Standard Penetration Tests (SPTs), particularly in Latin America. In this context, the use of commercial energy measurement devices has been essential in improving the accuracy of SPT blow count values, thereby enhancing the reliability of geotechnical designs based on these tests.

Various devices with similar objectives are being developed noncommercially. These devices have an academic nature, providing full control over the equipment and acquired signals, thus facilitating further research. Notable references include patents from Korea University with the code KR102371170B1 (Lee, J et al.) [22], which identified an increase in energy loss as the rod length increases. Additionally, the patent CN217325297U, titled “Testing Device for Measuring Energy Generated by Dynamic Penetration Hammering”, developed in China, incorporates sensors near the tip of the rod to accurately measure the energy absorbed by the rod (Sun Miaojun et al.) [23].

In Peru, issues arise regarding the accuracy of dynamic measurements of the SPT rod. While the Peruvian standard (NTP 399.133) [24] addresses aspects such as procedures and sampler types, the lack of equipment standardization complicates the use of the obtained geotechnical parameters. There is no recommendation for a Peruvian standard or code that measures energy during the execution of the SPT.

Despite advances in the development of instruments and equipment to address the lack of standardization in the Standard Penetration Test (SPT) in Peru, no substantial effort has been made to establish energy measurement-based standardization or to implement a reference procedure. In this context, the Digital Transformation Center at the National University of Engineering has taken a proactive approach by conducting comprehensive energy measurements using the Pile Dynamics PDA system at various locations within its territory. This initiative aims to gain a thorough understanding of the penetration test results, recognizing the need for detailed and reliable data.

Additionally, in response to the lack of standardization, the center has developed its own low-cost device called the Kallpa. This device not only accurately measures energy but is also designed to guide future regulations and contribute to the standardization of SPT results in Peru. This integrated approach aims to improve the consistency and reliability of penetration test results in Peru, laying the foundation for more uniform and robust practices in the future.

## 2. Methods and Materials

### 2.1. The Standard Penetration Test

The Standard Penetration Test (SPT) is an in situ geotechnical investigation method. This procedure involves driving a standardized split-barrel sampler into the soil under controlled conditions and recording the number of hammer blows required to achieve a penetration of 45 cm.

The Dynamic Penetration Test is one of the fundamental in situ techniques in geotechnical engineering. This method involves applying a specific energy using a hammer to drive a probe of defined dimensions into the soil, assessing its mechanical properties based on penetration resistance. It is particularly effective for granular soils that are challenging to sample and difficult to penetrate using static methods.

### 2.2. Energy Calculation

The evaluation of the energy generated by a hammer has evolved since the early experiments conducted in the 1970s by Schmertmann and Palacios [12]. These pioneers carried out some of the first measurements of hammer energy and applied one-dimensional wave mechanics, similar to those used in pile driving analysis, to investigate the Standard Penetration Test (SPT). At that time, the poor quality of accelerometers available led Schmertmann and Palacios [11] to adopt a force-based approach for energy measurement.

It was not until 1997 that Abou-Matar and Goble [25] introduced an innovative approach by using instrumentation that combined force and velocity, improving the energy measurement method and overcoming earlier limitations. In any case, the energy transmitted by the dynamic impact of an SPT hammer can be described as the work performed by the falling mass of the SPT hammer system.

W = ∫ Fdx(1)

where W is the work, F is the force, and dx is the incremental distance. Expressed as a function of time, the work formula describes the energy transferred as a function of force and velocity:

(2) $$W(t)=E(t)=\int 〖F(t)\frac{dx}{dt}dt=\int F(t)v(t)dt〗$$

where F(t) is the force in the rod as a function of time, v(t) is the particle velocity of the rod as a function of time, and E(t) is the energy transferred. The energy transferred from an SPT hammer impact is equal to the definite integral:

E(t) = ∫F(t)v(t)dt(3)

When integrated, the maximum energy transferred to the drill string can be determined.

Instead of directly measuring force and velocity, it is more practical to measure strain and acceleration. Accordingly, the following Equations (4) and (5) are used to compute force and velocity from the energy Equation (3).

F = εEA(4)

V = ∫a dt(5)

where ε is the strain measured on the drill rod, a is the acceleration measured on the drill rod, E is the modulus of elasticity of the drill rod at the sensor location, and A is the cross-sectional area of the drill rod at the sensor location.

The tests were conducted using the Pile Driving Analyzer, a signal processing and conditioning device that records and stores acceleration and strain data for each hammer blow during the Standard Penetration Test (SPT). This equipment interprets the measured signals generated by the propagating stress wave and, in real time, integrates the acceleration signals to determine particle velocity. Furthermore, it calculates force from the measured strain signals using Equation (4). The raw voltage signals from each transducer are converted into engineering units using calibration factors provided by the manufacturer. The maximum transferred energy is calculated using Equation (3).

**Figure 1:**
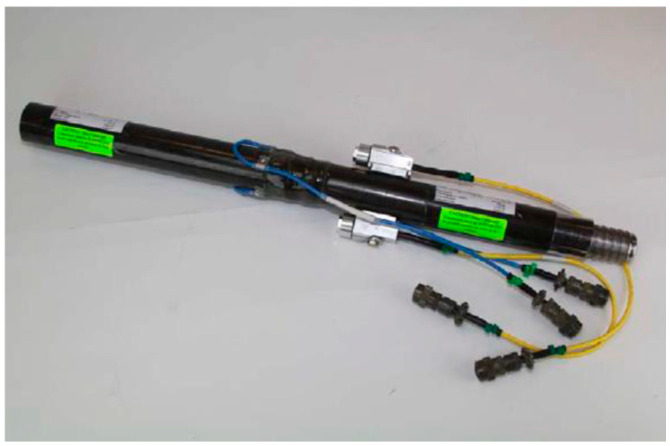
Instrumented rod for energy measurement.

During the test, force and velocity measurements are obtained using strain gauges and accelerometers installed on a 2-foot-long drill rod subassembly. This subassembly, illustrated in Figure 1, is positioned at the top of the drill rod string, beneath the anvil. Consequently, the measurements are taken at the top of the SPT system rather than at the sampler. The transferred energy is measured and averaged over the hammer impacts, which are then used to calculate the N value at a specific sampling depth during the SPT.

According to ASTM D4633, at least three to five energy–depth measurements are typically conducted in a single soil boring and averaged to obtain an overall energy measurement for the SPT hammer. This average measured energy is commonly referred to as the “calibration” of the SPT hammer. In practice, the transferred energy measured by the SPT hammer is divided by the theoretical maximum potential energy, which is 0.48 kN-m (4200 in-lb), and expressed as a percentage to quantify (Equation (6)) what is known as the Energy Transfer Ratio (*ETR*).

(6) $$ETR=\frac{E_{Actual}}{E_{Theoretical}}(100\%)$$

### 2.3. Testing Procedure—Peru Case

The Digital Transformation Center in Engineering (CITDI) conducted a series of studies across various regions in Peru to assess the performance of Standard Penetration Test (SPT) hammers available in the local market through energy measurements. During the penetration testing, the energy transmitted by the hammers was measured in accordance with the protocols specified in ASTM D1586 [1] and ASTM D4633 [21] standards. The SPTs were performed by multiple geotechnical firms employing both manual and automatic hammer release mechanisms across a variety of soil conditions. Concurrently, the CITDI monitored the hammer’s energy transfer during the free fall using different equipment employed by these geotechnical firms.

**Figure 2:**
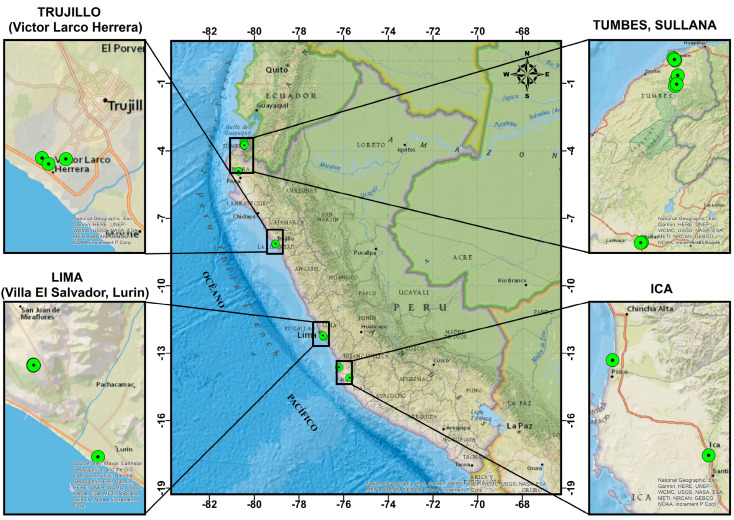
Location of energy measurement tests conducted by the Digital Transformation Center in Peru.

As illustrated in Figure 2, the tests were conducted in five cities, Tumbes, Pisco, Trujillo, Ica, and Villa El Salvador, Lima, over the period from 2021 to 2023. The majority of the hammers used had a nominal weight of 63.5 kg (140 lbs). Sixteen standard penetration tests were performed using donut-type hammers with a manual lift-and-release mechanism, along with two tests utilizing automatic hammers. Table 1 provides detailed information such as the year of execution, location, hammer type, hammer lifting/release mechanism, and the maximum depth achieved during each test.

### 2.4. Instruments and Testing Equipment

**Figure 3:**
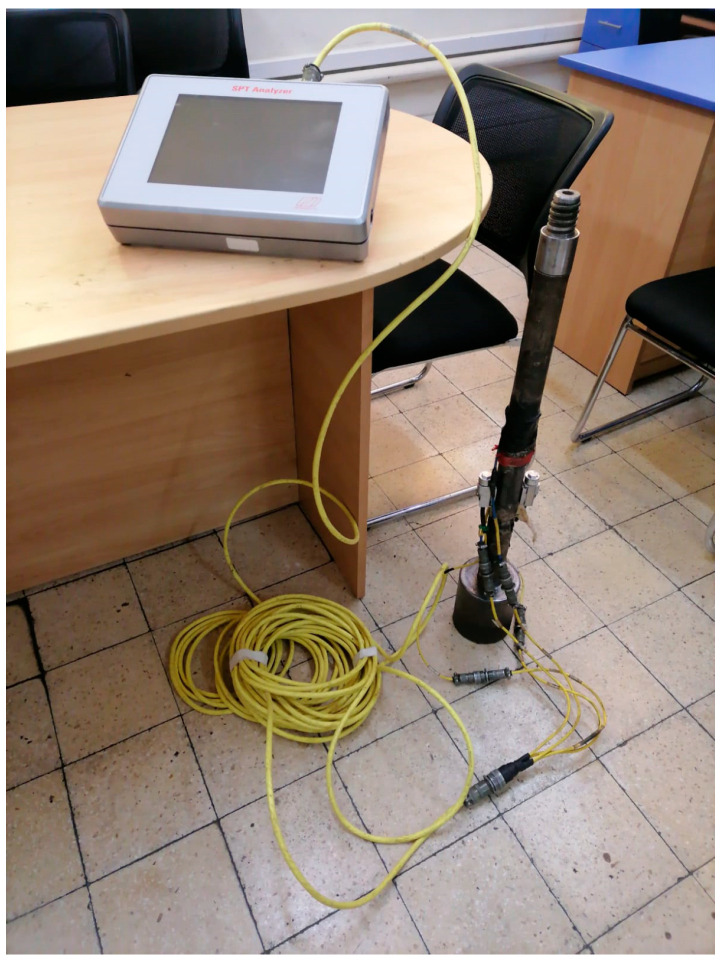
Pile Dynamics measurement equipment, where the accelerometers and strain gauges are directly connected to the instrumented rod section.

SPT energy measurements were conducted using the Kallpa SPT analyzers and devices from Pile Dynamics. These instruments record data obtained from an instrumented rod equipped with acceleration and strain sensors. The SPT Analyzer collects information from these sensors and processes the signals generated after each hammer blow during the execution of the SPT. Additionally, the PDA-S software analyzes the recorded files for interpretation, thereby determining the energy transferred to the SPT rod. Figure 3 shows the measurement equipment from Pile Dynamics.

## 3. Development of the Kallpa Device

Standard Penetration Tests (SPTs) are the most commonly used methods for determining soil characteristics and properties. However, these tests are somewhat imprecise, requiring corrections for proper data interpretation. One of these corrections is the energy correction, which involves calculating the actual energy absorbed by the rod to determine the test’s energy efficiency.

Currently, there are few devices available for energy measurement in Standard Penetration Tests. This is especially true in Latin America, where one reason for this is the lack of standardization in energy measurement during SPT testing. North American and European standards indicate that this correction is necessary due to the variety of methods and techniques used for conducting the SPT.

A globally recognized commercial device is the PDA analyzer by Pile Dynamics. This is a four-channel device capable of sampling at a 16-bit resolution with a 100 kHz sampling frequency per channel. However, the analyzer has dimensions of 320 mm × 250 mm × 68 mm and weighs 5 kg, which makes it somewhat cumbersome and difficult to transport. However, this analyzer complies with European standards for energy measurement in penetration tests because it has a sampling frequency of no less than 100 kHz.

According to ASTM D4633 [21], for calculating energy in SPT tests, a minimum sampling frequency of 50 kHz is sufficient. This frequency allows capturing the essential dynamic response of the system. However, higher sampling frequencies, such as 100 kHz or 150 kHz, provide a better resolution to detect rapid force variations, improving the accuracy in signal interpretation and energy efficiency determination.

In Peru, the lack of locally manufactured specific equipment has contributed to the high costs of calibrating SPT tests. This article also details the development of an energy measurement device called “Kallpa”. This innovative device uses low-cost electronic components for digitalizing the signals from the sensors used in SPT tests.

### 3.1. Design

**Figure 4:**
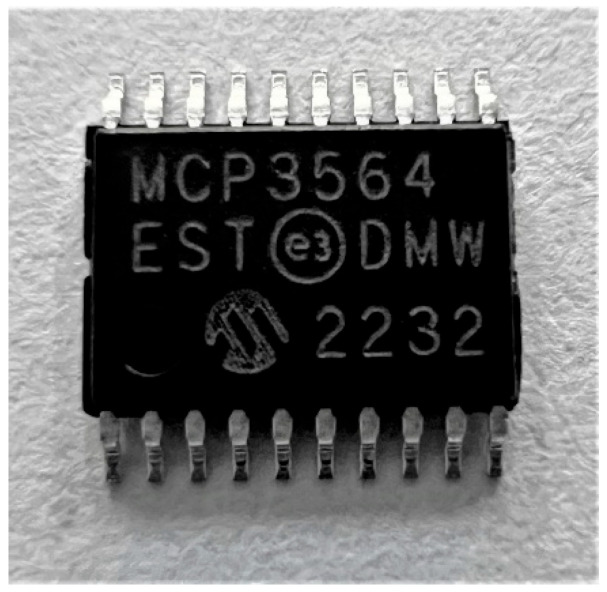
MCP3564 analog-to-digital converter.

Taking into account the minimum requirements for an energy analyzer (ASTM D4633)) [20], an analog-to-digital converter (ADC) was used (Figure 4). This converter can sample at 153 ksps and has a 24-bit resolution. As seen, it meets the minimum specifications outlined in the ASTM D4633 standard. Additionally, it can register up to eight channels, which is sufficient for the four required sensors (two channels per sensor). However, an independent ADC was used for each sensor. This decision was made because using a single ADC for all four sensors (two strain gauges and two accelerometers) would have limited the sampling frequency. When a single ADC was used, the overall system sampling frequency dropped below 50 kHz, which did not meet the established requirements for analysis.

A Raspberry Pi microcomputer was employed for reading and storing data from the analog-to-digital converters (ADCs). The Raspberry Pi 4, a low-cost, compact board, is widely used in electronics, robotics, home automation, and numerous other applications. In this project, the Raspberry Pi 4 communicates with the ADCs via the Serial Peripheral Interface (SPI) protocol, facilitated by the “pigpio.h” library and custom C code.

**Figure 5:**
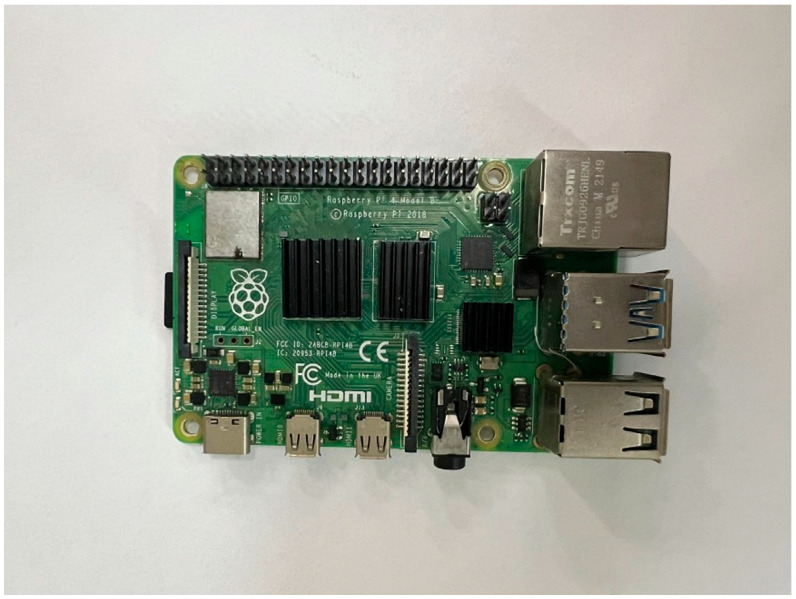
View of the Raspberry Pi 4.

With this program, the Raspberry Pi 4 can simultaneously sample data from four sensors at a sampling rate of up to 100 ksps with a 16-bit resolution. This enables accurate, real-time data logging during Standard Penetration Test (SPT) procedures. Additionally, using the Raspberry Pi 4 as a microcontroller offers several advantages, including low cost, ease of use and programming, and the ability to connect to various devices and peripherals (Figure 5).

**Figure 6:**
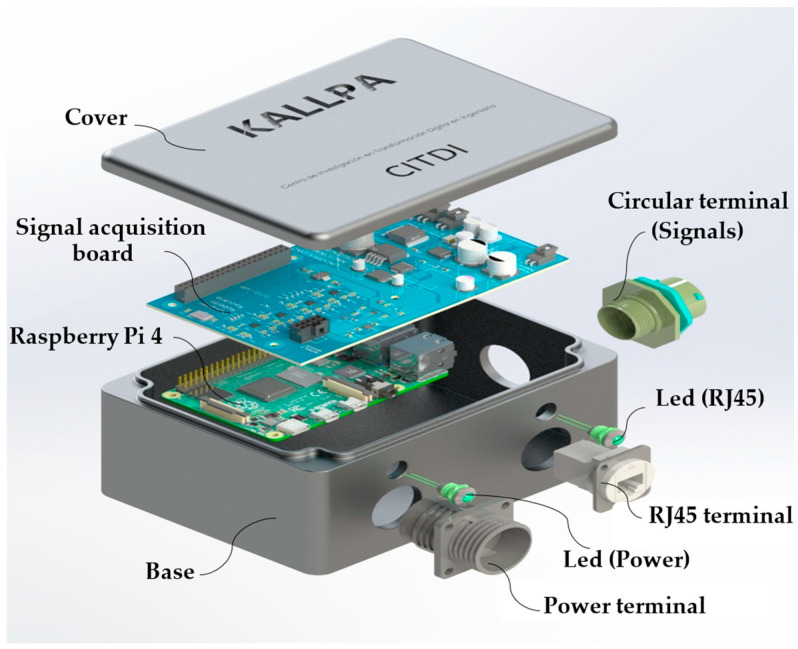
Components of the Kallpa measurement equipment.

As depicted in Figure 6, the device comprises eight primary components: a base, a lid, an electronic board, a data acquisition board, a 19-pin circular connector, an RJ45 connector, a 4-pin circular connector, and two LED indicators. Housed within a protective enclosure are an electronic board, a power regulation module, a signal acquisition module, and two LED indicators. The primary function of this enclosure is to safeguard these modules against external environmental influences. The LED indicators, positioned at the base of the enclosure, provide a visual indication of the device’s power status.

The control module is secured to the lower portion of the enclosure using four bolts. The signal acquisition module and power supply module are integrated into the data acquisition board, which interfaces with the electronic board via General Purpose Input/Output (GPIO) pins. The signal acquisition board is situated above the control module. Additionally, the signal acquisition board features a molex connector to which the 19-pin circular connector is attached.

### 3.2. Data Acquisition System

In order to correctly acquire data from the ADC converters, the use of low-pass filters on the signal inputs is necessary. These filters allow low-frequency signals to pass through while attenuating or eliminating high-frequency signals. Their primary function is to reduce noise and high-frequency interference that could affect the quality of the sensor signals. The ASTM D4633 [21] standard specifies a minimum filter frequency of 5 kHz.

**Figure 7:**
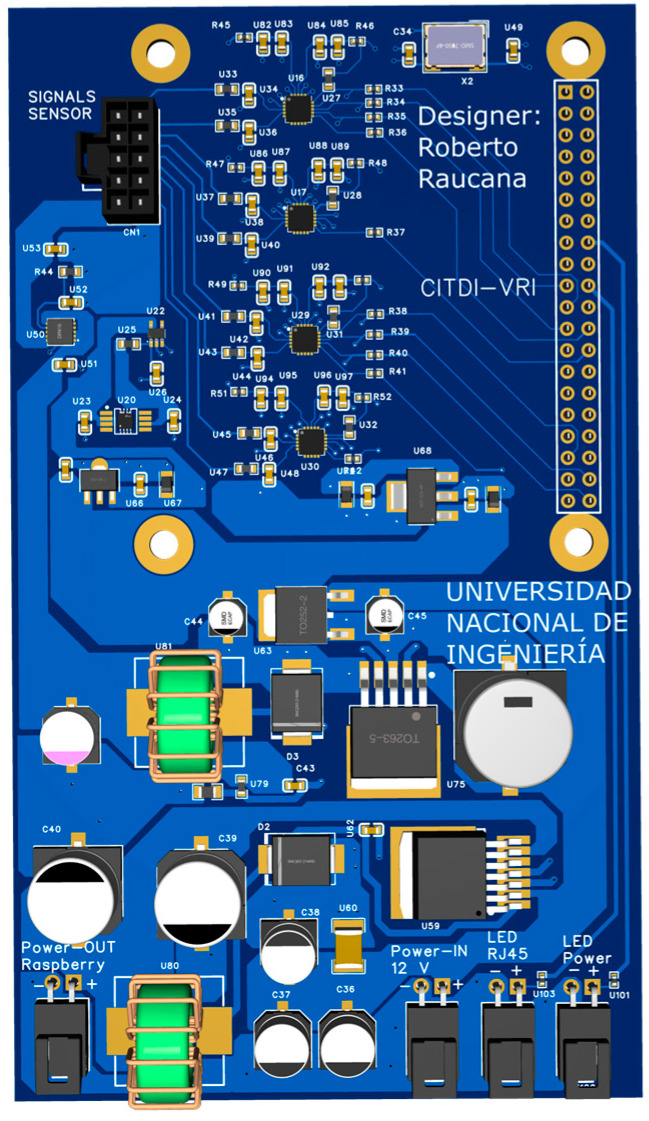
Design of the signal acquisition board.

Therefore, first-order RC (resistance/capacitance) causal filters were used on the PCB (Printed Circuit Board) to build the data acquisition board (Figure 6). Among the most important components of this board are the ADCs, which were soldered along with the RC filters, composed of a resistor and a capacitor, and the precision 3.3 V voltage regulators that supply the precise input voltage required by the ADCs. As previously mentioned, this data acquisition system can sample all four sensors simultaneously at 100 kHz with a 16-bit resolution. The maximum resolution of the MCP3564 ADC is 24 bits; however, when the ADC is configured to this resolution, the sampling frequency drops below 50 kHz. For this reason, the ADC was configured to operate at a 16-bit resolution. Figure 7 shows the flow diagram of the Kallpa device.

**Figure 8:**
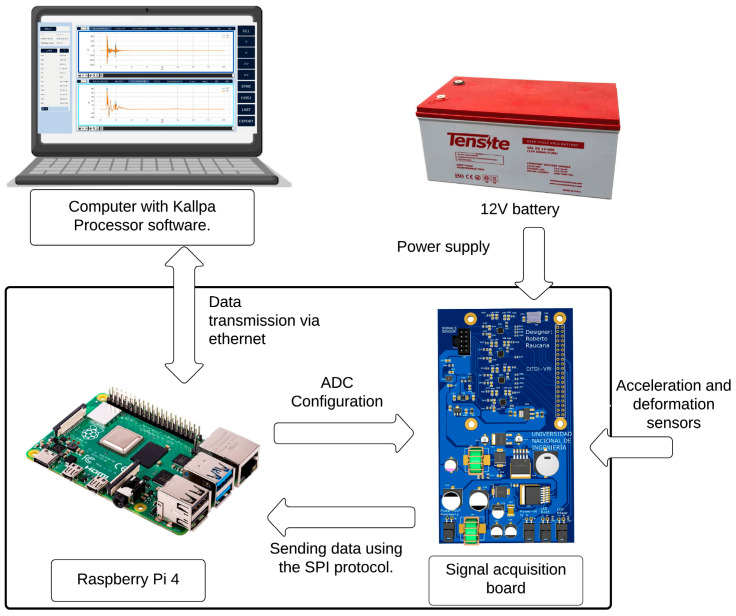
Flow diagram of the Kallpa device.

Figure 8 illustrates the connection flow within the Kallpa device. The system is powered by a 12 V battery, which supplies power to the signal acquisition board. This board is responsible for distributing power to both the ADCs and the Raspberry Pi. In addition, sensor signals enter through the signal acquisition board, where they undergo filtering to remove high-frequency noise before being digitized by the ADCs. The digitized signals are then transmitted to the Raspberry Pi, which processes the data in real time. The Raspberry Pi continuously monitors the incoming signals for impact events. When an impact is identified, the processed information is immediately transmitted via an Ethernet connection to a computer running the Kallpa Processor software, where it is stored for further analysis.

### 3.3. Kallpa Processor Software

The Kallpa Processor program is designed to process the data obtained from the hammer drop test. It was developed in Python (Version 3.10.14). For interaction with the Kallpa Processor program, a user-friendly and intuitive interface has been created, utilizing a modern version of the “tkinter” library called “customtkinter,” which includes various visual enhancements. This library allows for customization of element styles similar to CSS and adjusting color schemes according to the default Windows theme.

**Figure 9:**
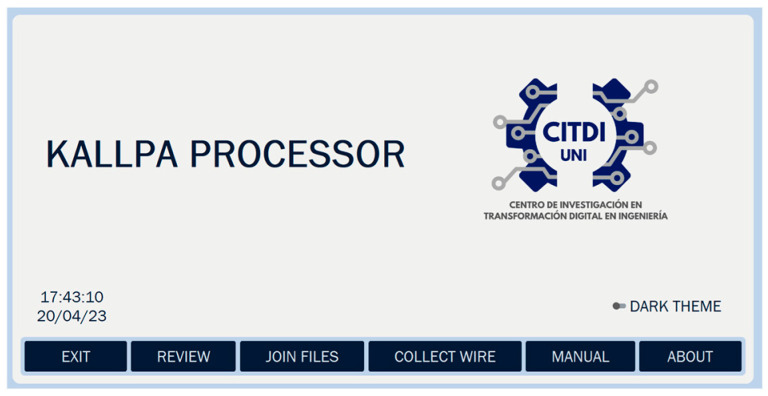
Kallpa Processor software startup interface.

The program enables the user to quickly access three key functions: Join Files, Collect Wire, and Review, each focused on specific tasks related to data processing and analysis (Figure 9).

The Join Files function is responsible for converting files from the “.csv” format to a “.ct” format. The latter is compatible with the software. To achieve this, data obtained, such as acceleration and deformation data, must first be saved in a CSV file. Then, the user configures the sampling parameters necessary to ensure proper conversion of the data into the correct format. This process ensures that the CSV files are properly transformed into “.ct” files that can be opened and viewed without errors within the program interface.

On the other hand, the Collect Wire function enables real-time data collection from the Kallpa device. This function connects to the device and displays the information immediately on the program interface. As the data are collected, they are processed, and once the collection is complete, a new “.ct” file is generated containing all the captured data. This function is crucial for monitoring and recording real-time data during testing.

Additionally, the Review function aims to process and analyze the data obtained from the sensors. This tool allows the user to calculate and view detailed information about deformations, forces, displacements, velocities, and energies generated during the data collection process. It also enables the visualization of recorded impacts, facilitating the interpretation of experimental results and the generation of a PDF report on the test.

**Figure 10:**
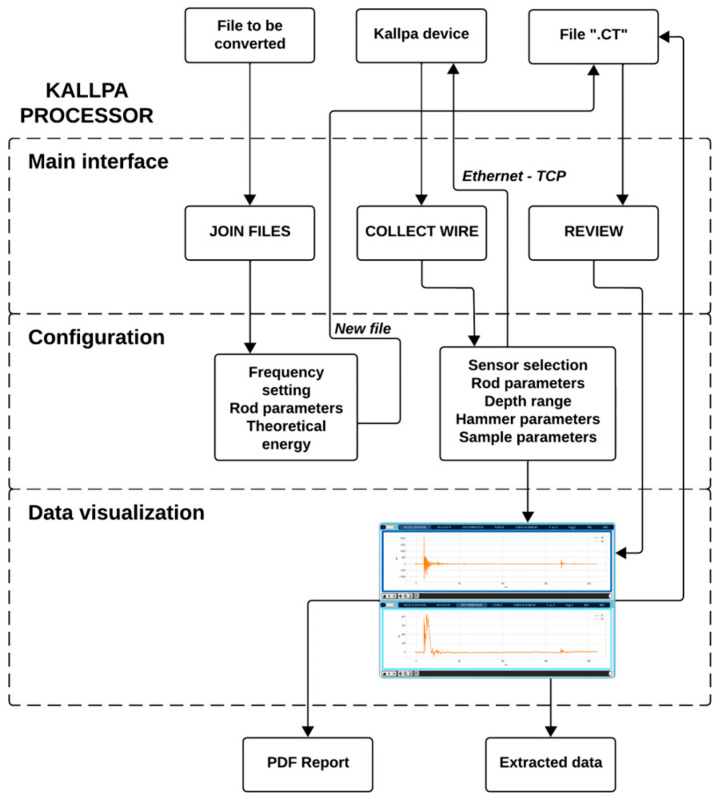
Functional architecture of Kallpa Processor.

Figure 10 illustrates the functional architecture of the software, showing how these three functions interrelate to provide an efficient and effective workflow. Through these functionalities, the Kallpa Processor software provides a robust and versatile platform for processing data from the Kallpa measurement device, ensuring correct interpretation and analysis.

#### 3.3.1. Information Collection

**Figure 11:**
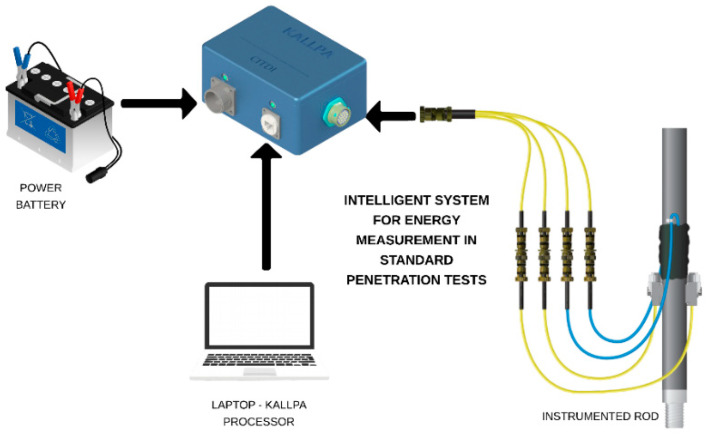
Connection diagram of the Kallpa device.

For information collection, the Kallpa device must be connected as shown in the diagram in Figure 11. The sensors located on the SPT rod must be connected to the Kallpa device using a 19-pin circular connector. This device can be powered in two ways: either by a 12-volt battery or by a transformer that converts the 220 V AC signal to 12 V, in case it is connected to a 220 V AC power source. Additionally, the Kallpa device is connected to a laptop via an Ethernet cable, allowing it to interact with the Kallpa Processor program for data management and processing.

**Figure 12:**
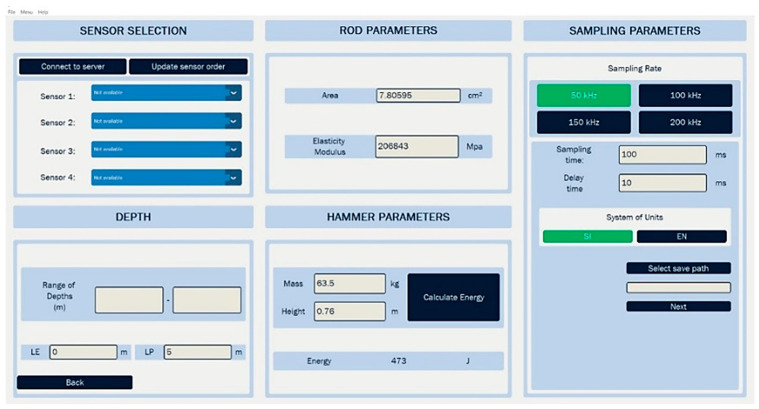
Parameter configuration of the Collect Wire window.

Once the connection is established, the next step is to configure the parameters in the “Collect Wire” window, as shown in Figure 12. In this window, the sensors to be used are selected, the corresponding depth ranges for each measurement are entered, and the sampling parameters are adjusted, including the time and sampling frequency. Additionally, specific parameters for the rod must be entered, such as its area and elasticity modulus, as well as the hammer parameters, which include mass and drop height. This configuration ensures that all data collected during the test are aligned with the technical requirements of the test.

With all the parameters correctly configured, data collection begins, along with real-time visualization. This allows continuous monitoring of the test’s progress and the ability to adjust if necessary. Upon completion of the test, the Kallpa Processor program generates a file with the “.ct” extension that contains all the information obtained throughout the test, enabling a detailed post-test analysis of the collected data.

#### 3.3.2. Information Visualization

**Figure 13:**
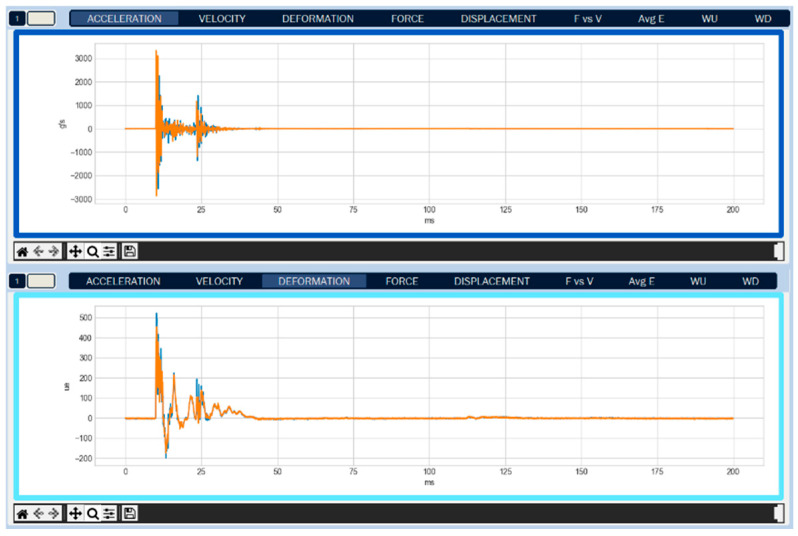
Visualization of two acceleration signals and to deformation signals from the test carried out at UNTELS (Kallpa Processor—Version 1.0). The graph shows two sensors per magnitude, differentiated by the orange and blue colors.

The Kallpa Processor software allows the operator to view various real-time graphs. These include acceleration, velocity, displacement, deformation, force, and energy (Figure 13). This facilitates test supervision and the detection of any anomalies or errors in the process.

The program displays two graphs simultaneously. Each graph can represent different magnitudes, such as acceleration, velocity, or force, and may correspond to the same impact or different impacts. For example, the top graph could show the energy of impact number 4, while the bottom graph shows the energy of impact number 10. This functionality allows for comparing the graphs between different impacts.

**Figure 14:**
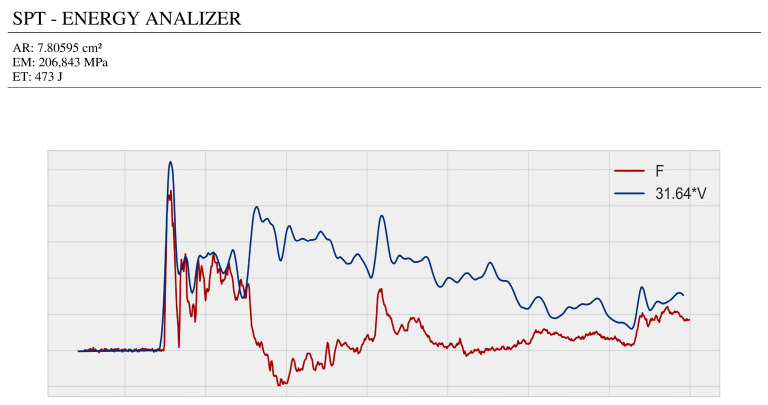
Top of the PDF report generated by the Kallpa Processor software: force–velocity graph.

**Figure 15:**
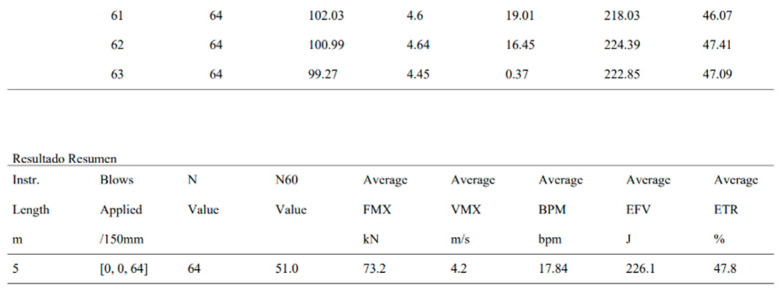
Bottom of the PDF report generated by the Kallpa Processor software: summary of energy data from the test.

For data export, the software offers two main formats: PDF and Excel. When exporting to PDF, the “fpdf2” library is used, which allows for generating a test report in PDF format (Figure 14 and Figure 15). This report includes a force–velocity graph for a test impact, a table with the maximum values of the most important magnitudes, a summary of these values, and the number of blows calibrated by energy (N60). In Excel format, the “xlsxwriter” library is used, which is lighter and has fewer features than other libraries like “pandas”. The Excel file contains all the data for the measured magnitudes, such as acceleration, deformation, force, displacement, velocity, and energy.

## 4. Results

### 4.1. Kallpa Equipment—Calibration

To calibrate the sensor data, the PDA analyzer was used. This energy meter was developed by Pile Dynamics. The sensors were connected in parallel, with one pair consisting of an accelerometer and a deformation gauge connected to the PDA analyzer, and the other connected to Kallpa.

For calibration, approximately 500 impacts were used, and the maximum values from each device were compared. The Kallpa device data are in microvolts (µV), while the SPT Analyzer data are expressed in gravities (g’s) and microstrains (µε). The average of each data set was calculated, and the calibration factor was determined as the ratio between these averages. Linear regression graphs were also generated with the coefficient of determination (R^2^) to assess the quality of the fit and the reliability of the calibration factors.

**Figure 16:**
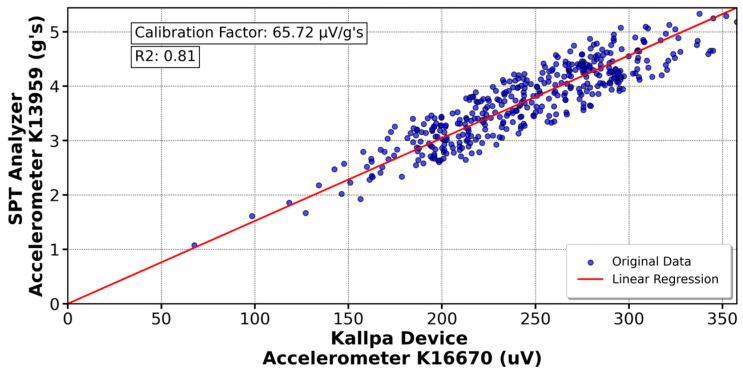
Calibration of the K16670 accelerometer in the Kallpa device compared to the PDA analyzer.

**Figure 17:**
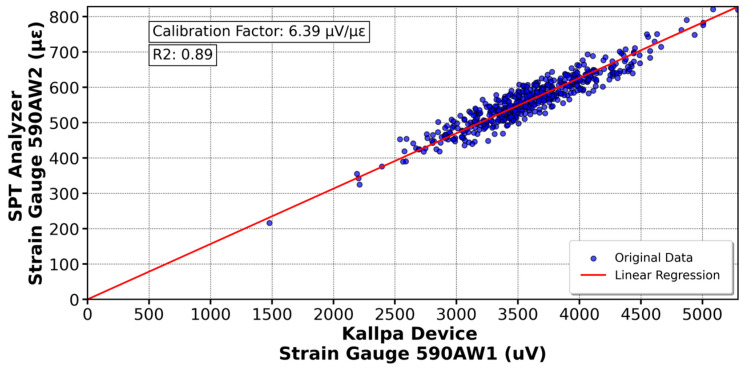
Calibration of the strain gauge 590AW1 in the Kallpa device compared to the PDA analyzer.

**Figure 18:**
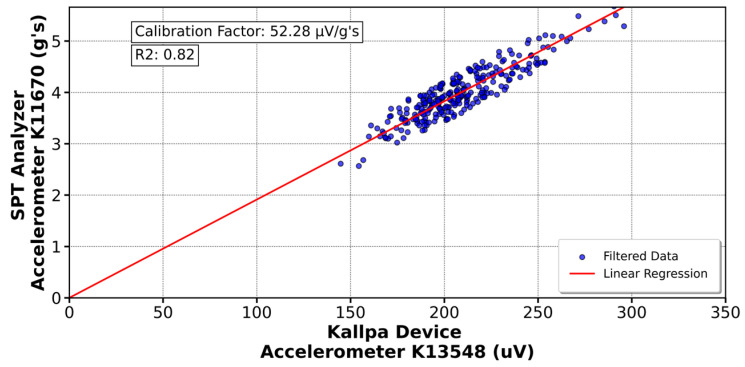
Calibration of the K13548 accelerometer in the Kallpa device compared to the PDA analyzer.

**Figure 19:**
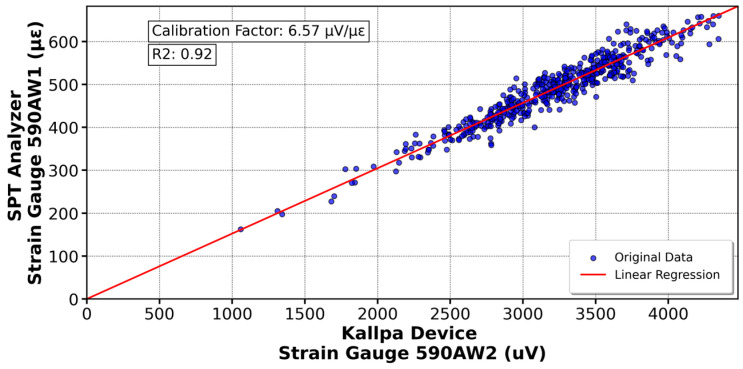
Calibration of the strain gauge 590AW2 in the Kallpa device compared to the PDA analyzer.

Figure 16 shows the calibration of the K11670 accelerometer (Kallpa) compared to the K13959 sensor (SPT Analyzer), resulting in a calibration factor of 65.72 µV/g’s. Similar analyses were conducted with other sensors. In Figure 17, the 590AW1 deformation gauge (Kallpa) was calibrated against the 590AW (SPT Analyzer), yielding a factor of 6.39 µV/µε. For the sensors shown in Figure 18 and Figure 19, calibration factors of 52.28 µV/g’s for the K13548 accelerometer and 6.57 µV/µε for the 590AW2 deformation gauge were obtained.

**Figure 20:**
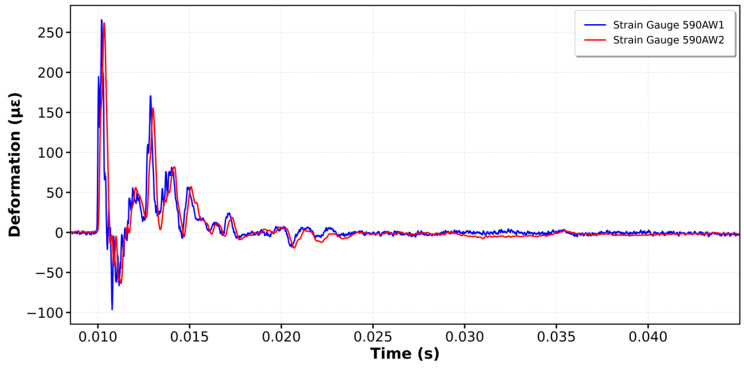
Comparison impacts between Pile and Kallpa: strain gauge impact.

**Figure 21:**
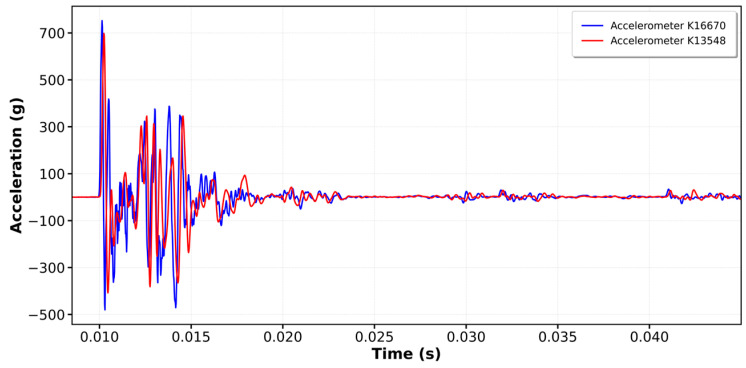
Comparison impacts between Pile and Kallpa: acceleration impact.

Figure 20 and Figure 21 show the graphs from Kallpa and Pile Dynamics for some impacts. Considering that the Kallpa graphs already have the conversion factor applied, a good correlation and similar shapes can be observed.

### 4.2. Energy Measurement Results

In Peru, the number of manual donut hammers significantly exceeds that of automatic hammers. This study analyzed the Energy Transfer Ratio (ETR) of SPT hammers. A total of 14 manual-release donut-type hammers were used, with a database containing 3430 impact energy records transmitted to the rods, along with 2 automatic hammers that recorded 465 energy transmission impacts. This disparity in the number of devices used reflects the predominance of manual hammers in Peru and explains the imbalance in the sample.

According to the figures presented in Table 2, it can be observed that the manual donut-type hammers exhibited ETRs ranging from 43.5% to 68.4%, with an average of 58.9%. On the other hand, the automatic hammers showed ETRs of 82% and 87%. These results are consistent with previous studies, such as Schmertmann [11], which indicate that the ETRs of manual donut hammers tend to be lower than those of automatic hammers due to variations in hammer efficiency and energy dissipation.

With this information, the corrections for the number of blows in the 16 Standard Penetration Tests (SPTs) were made. Additionally, adjustments were made for borehole diameter, drill rod length, and sampler type. No correction for the water table was applied, as no groundwater was identified in the study area.

**Figure 22:**
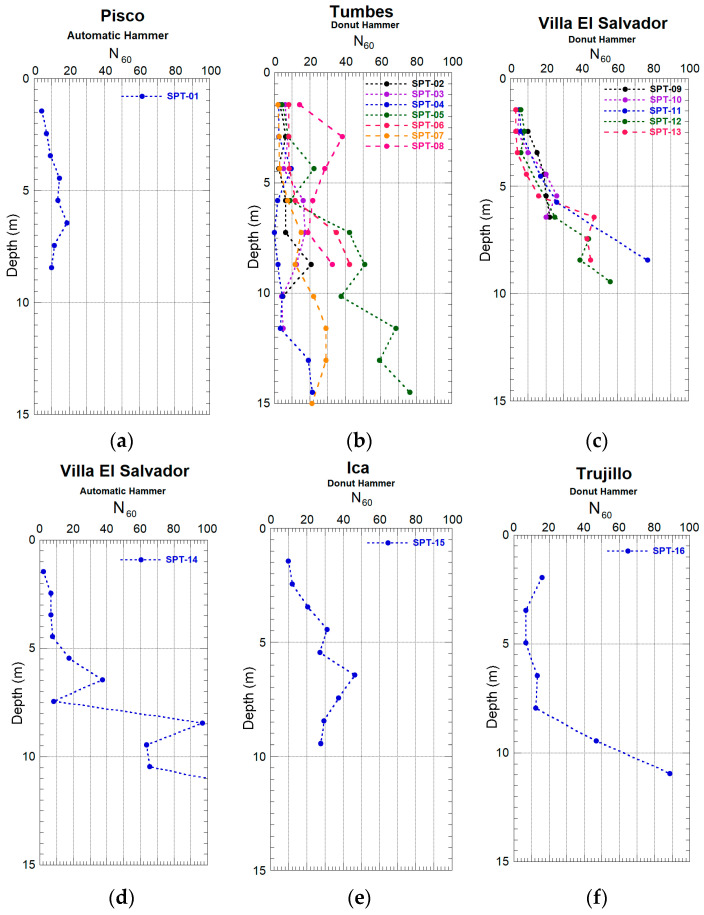
Measured ETR (%) values as a function of depth for manual and automatic donut-type hammers. Pisco (**a**), Tumbes (**b**), Villa El Salvador manual equipment (**c**), Villa El Salvador automatic equipment (**d**), Ica (**e**), and Trujillo (**f**).

After applying these corrections, the N₆₀ value was obtained and correlated with the relative density of the soil. Based on this correlation, the soils in the study area were classified into very loose, loose, medium dense, dense, or very dense categories, allowing for a more accurate geotechnical interpretation of the site conditions. The tests were conducted in sandy soils, and the results are presented in Figure 22a–f. These results reveal significant differences in the soil properties across the evaluated locations.

## 5. Discussion

### 5.1. Variability in the Relative Density of Sands According to the SPT Test Results

The correction of the N_60_ blow count allowed for the estimation of the relative density of the evaluated soils. For the soils in Villa El Salvador, the N60 value, obtained through Standard Penetration Tests (SPTs), increases significantly from a depth of 7 m. In contrast, tests performed in Tumbes, Pisco, and Ica do not show this trend, whereas in Trujillo, a similar behavior to that recorded in Villa El Salvador is observed.

In the Pisco area, SPT tests indicate that the sands are moderately dense from 2 m to 8.45 m in depth, showing relative uniformity in terms of soil characteristics. On the other hand, in Tumbes, the results vary depending on the test. In SPT tests 02 and 04, the sands are classified as very loose to loose over the entire evaluated depth. In tests 03 and 07, the N60 values indicate that the sands are very loose to loose up to 5 m, whereas at greater depths, they become moderately dense. Tests 05 and 06 show loose sands up to 6 m, and dense to very dense sands at greater depths. In contrast, test 08 shows moderately dense to dense sands uniformly from 1 m to 9 m.

In the Villa El Salvador district, SPT tests 09, 10, and 12 indicate that the sands in the shallow layers are loose but become moderately dense to dense from 3 to 4 m deep. According to SPT test 11, the sands are moderately dense to dense up to 6 m and are classified as very dense at greater depths. Finally, tests 13 and 14 show loose sands up to 4 m, moderately dense sands up to 5 m, and very dense sands from this depth onwards.

In the Ica area, the SPT test from the first evaluation point reveals that the surface stratum consists of loose sands, whereas at greater depths, the strata are classified as moderately dense to dense. In Trujillo, the results from the SPT tests indicate that the upper strata, up to 8 m, consist of loose to moderately dense sands, whereas below 9 m, the sands are dense to very dense.

These results, which are based on the N60 values obtained from the SPT tests, show significant differences in the soil properties of the evaluated locations, which are influenced by the ground conditions, the testing method, and the operational procedure. Energy efficiency plays a key role in this variability, as the reliability of the N value depends on the ability to maintain constant energy delivery during the test. Researchers such as Gibbs and Holtz [26], Terzaghi and Peck [27], and Marcuson and Bieganousky [28], have worked on correlating the SPT results with soil properties such as relative density, shear strength, and friction angle. In this regard, a key aspect for optimizing the design of geotechnical structures is the precise monitoring of the energy delivered during the tests, which allows for a more accurate approximation of the obtained results.

### 5.2. Kallpa Equipment

For the Kallpa device, the coefficient of determination (R^2^) for the sensors is sufficiently close to 1, which validates the reliability of the collected and analyzed data. However, for the strain gauges, this value is even closer to 1, approximately 0.96. This difference is attributed to how the sensors are connected to the rod. While the strain gauges are embedded and firmly attached to the rod, the accelerometers are fixed with bolts. This results in a higher data reliability of the three strain gauges than the accelerometers when conducting the SPT test. As a comparison, the Kallpa device was used alongside the Pile Dynamics SPT Analyzer, and showed that the Energy Transfer Ratio (ETR) measurements differed by 3–5%, with Kallpa having lower values.

From the results, we can see that the Kallpa device offers advantages over other SPT systems due to its small, compact, and portable design, which facilitates its use in various field conditions. Additionally, its construction with low-cost electronic components makes it a more accessible option for geotechnical professionals. Despite its lower cost, Kallpa maintains functionality equivalent to that of more expensive energy analyzers. Its system is trained to automatically identify valid impact records and eliminate potential false positives, ensuring accurate energy measurements.

At the end of the test, the device, supported by the Kallpa Processor software, generates a detailed report that determines the average energy of all impacts recorded during the SPT. The equipment can be connected to any laptop, and once the data are stored in its internal registry, it transmits the information to a computer for storage and real-time visualization. This capability enables more efficient data analysis and improves the interpretation of test results.

### 5.3. Energy Measurement

**Figure 23:**
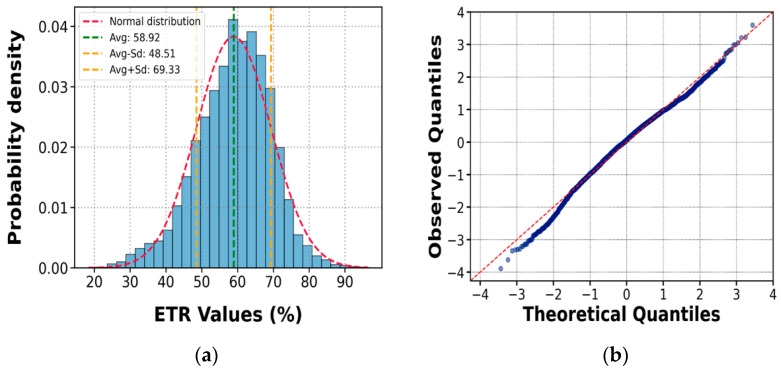
Plot for a normality test of ETR values for the manual SPT (**a**); Q-Q plot (**b**).

**Figure 24:**
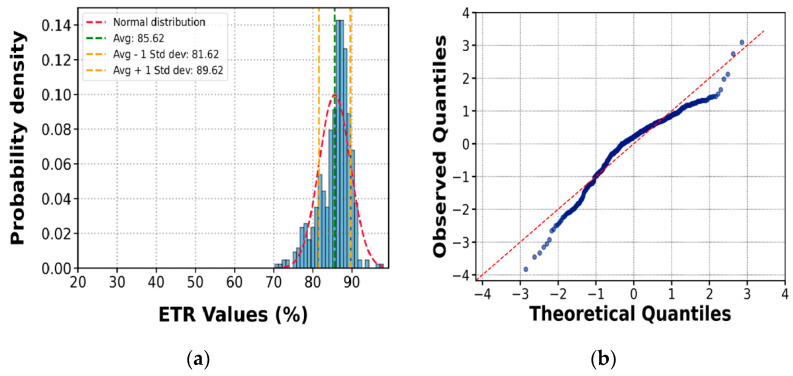
Plot for a normality test of ETR values for the automatic SPT (**a**); Q-Q plot (**b**).

For the analysis of energy measurement results, one of the most common graphical methods was used to evaluate the normality of the data. This involved constructing a histogram and overlaying the curve of a normal distribution with the same mean and standard deviation as the available data, as illustrated in Figure 23 and Figure 24 (for the manual and automatic equipment). Additionally, a quantile comparison plot, also known as a Q-Q plot, was used, indicating that the data more closely approximate a normal distribution.

The results from the 16 tests were analyzed according to hammer type, depth, soil type, and the number of blows (NSPT). The ETR and associated uncertainty values depend mainly on the impedance of the rods, weight, nonstandard drop height, and hammer release technique. From the values obtained in this study, the Energy Transfer Ratio (ETR) and the statistical uncertainty associated with these tests show that the manual ETR tends to an average of 58.9%, with a standard deviation of 10.4%, whereas the automatic ETR has an average of 85.5%, with a standard deviation of 4.2%. These results may be considered representative of the local practice of SPT testing in Peru.

**Figure 25:**
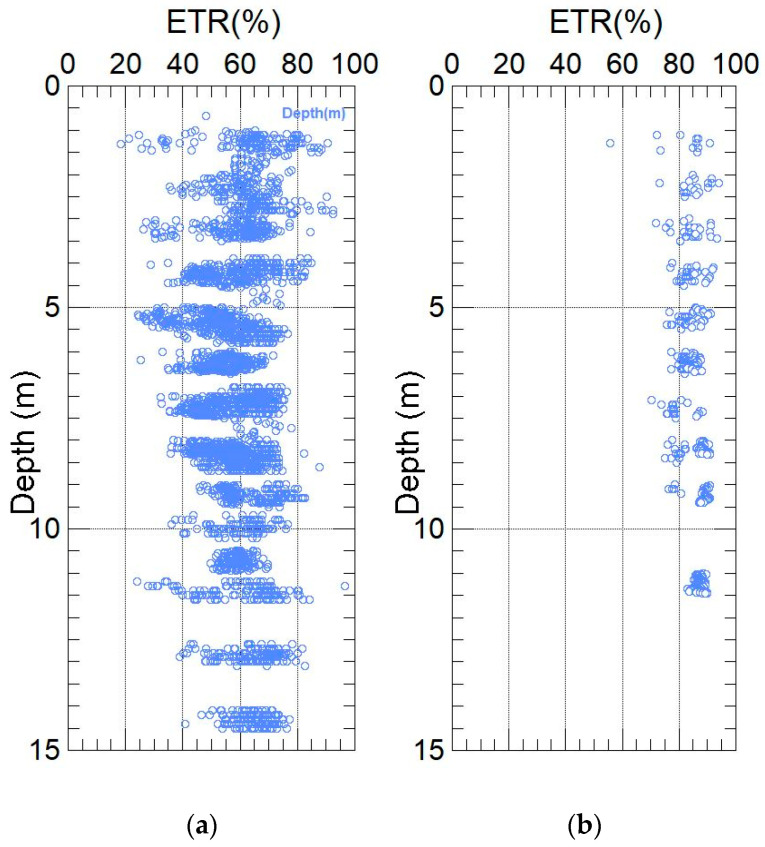
Measured ETR (%) values as a function of depth for manual donut hammers (**a**) and automatic hammers (**b**).

Moreover, at depths greater than 9 m, the energy values become more consistent for both the manual donut hammer and the automatic hammer. Figure 25 shows the combined distribution of the Energy Transfer Ratio (ETR%) values as a function of depth for the manual and automatic donut hammers.

## 6. Conclusions

This study evaluated the performance of the Kallpa device in energy measurement during Standard Penetration Test (SPT) tests and compared its results with those obtained from commercial devices such as the SPT Analyzer from Pile Dynamics. The data showed that Kallpa is reliable, with a determination coefficient close to unity for the sensors used. However, differences in precision were observed between the accelerometers and the extensometers, which was attributed to the way the sensors were connected to the rod. Compared with the commercial device, Kallpa has energy measurements (ETRs) that differ by 3–5%, with slightly lower values.

The analysis of soil relative density, which was based on the correction of the blow count (N_60_) obtained through the SPT tests, revealed significant variations between the studied locations. In Villa El Salvador, the N60 values increased from a depth of 7 m, whereas in Tumbes, Pisco, and Ica, this trend was not evident. In Trujillo, the results were similar to those in Villa El Salvador. These differences reflect soil heterogeneity and highlight the importance of considering both the N60 values and the local soil characteristics for interpretation.

Finally, the energy analysis revealed that the manual donut hammers presented an average ETR of 58.9%, with a standard deviation of 10.4%, whereas the automatic hammers presented an average ETR of 85.5%, with a standard deviation of 4.2%. Additionally, at depths greater than 9 m, the energy values were more consistent for both hammer types. These findings provide a framework for interpreting ETR values in the context of local SPT testing practices in Peru and validate the use of the Kallpa device as a viable alternative for energy and relative density measurements.

## 7. Patents

This work includes a patent application for the Kallpa device, filed in 2023. The equipment described in this manuscript is currently in the final stages of the patent process.

## Figures and Tables

**Table 1:** Hammer type and penetration depth with energy measurement.

Year	City	Test	Hammer Type	Measurement Depth (m)
2020	Pisco	SPT-01	Donut—Automatic	8.45
2021	Tumbes	SPT-02	Donut—Rope and Winch	10.15
		SPT-03	Donut—Rope and Winch	11.60
		SPT-04	Donut—Rope and Winch	14.50
		SPT-05	Donut—Rope and Winch	14.50
		SPT-06	Donut—Rope and Winch	8.70
		SPT-07	Donut—Rope and Winch	14.50
		SPT-08	Donut—Rope and Winch	7.25
2022	Villa El Salvador	SPT-09	Donut—Rope and Winch	5.45
		SPT-10	Donut—Rope and Winch	5.45
		SPT-11	Donut—Rope and Winch	8.45
2023	Villa El Salvador	SPT-12	Donut—Rope and Winch	9.45
		SPT-13	Donut—Rope and Winch	8.45
2023	Villa El Salvador	SPT-14	Donut—Automatic	11.45
2023	Ciudad de Ica	SPT-15	Donut—Rope and Winch	9.45
2023	Trujillo	SPT-16	Donut—Rope and Winch	10.95

**Table 2:** Statistical results of energy measurement for the executed tests.

Test Point	Energy Transfer Ratio (ETR)				Hammer Type
	Avg.	Mean + Standard Deviation	Mean – Standard Deviation	Standard Deviation	
SPT-01	82.0	78.1	85.8	3.9	Donut—Automatic
SPT-02	56.6	47.9	65.3	8.7	Donut—Rope and Winch
SPT-03	60.7	55.1	66.2	5.5	Donut—Rope and Winch
SPT-04	67.5	60.4	74.6	7.1	Donut—Rope and Winch
SPT-05	68.4	65.3	74.5	6.0	Donut—Rope and Winch
SPT-06	59.6	54.1	65.0	5.5	Donut—Rope and Winch
SPT-07	52.7	41.7	63.7	11.0	Donut—Rope and Winch
SPT-08	66.6	61.0	72.3	5.6	Donut—Rope and Winch
SPT-09	52.7	45.3	60.1	7.4	Donut—Rope and Winch
SPT-10	56.9	49.5	64.3	7.4	Donut—Rope and Winch
SPT-11	64.7	57.6	71.9	7.2	Donut—Rope and Winch
SPT-12	52.5	44.6	60.4	7.9	Donut—Rope and Winch
SPT-13	43.5	34.8	52.1	8.6	Donut—Rope and Winch
SPT-14	87.4	84.1	90.7	3.3	Donut—Automatic
SPT-15	56.4	50.0	62.8	6.4	Donut—Rope and Winch
SPT-16	60.5	54.5	66.5	6.0	Donut—Rope and Winch

## Data Availability

The data presented in this study are available from the corresponding author upon reasonable request due to privacy considerations.
